# Home Hazard Removal to Reduce Falls Among Community-Dwelling Older Adults: A Randomized Clinical Trial

**DOI:** 10.1001/jamanetworkopen.2021.22044

**Published:** 2021-08-31

**Authors:** Susan Stark, Marian Keglovits, Emily Somerville, Yi-Ling Hu, Abigail Barker, Dave Sykora, Yan Yan

**Affiliations:** 1 Program in Occupational Therapy, Washington University School of Medicine in St Louis, St Louis, Missouri; 2 Institute of Gerontology, Wayne State University, Detroit, Michigan; 3 Brown School of Social Work, Washington University in St Louis, St Louis, Missouri; 4 St Louis Area Agency on Aging, St Louis, Missouri; 5 Department of Surgery, Washington University School of Medicine in St Louis, St Louis, Missouri

## Abstract

**Importance:**

Falls are the leading preventable cause of morbidity, mortality, and premature institutionalization for community-dwelling older adults.

**Objective:**

To test the effectiveness of a behavioral intervention on fall risk among older adults receiving services from an Area Agency on Aging.

**Design, Setting, and Participants:**

This randomized clinical trial examined a home hazard removal intervention in the community using a race- and sex-stratified randomization design. Older adults receiving services from the Area Agency on Aging in urban St Louis, Missouri, were assigned to a home hazard removal intervention delivered over 2 weeks with a 6-month booster or usual care control. Eligible participants were adults aged 65 years or older who did not have dementia, were at high risk for falling, and resided in the community. Enrollment occurred from January 2015 to September 2016; 12-month follow-up occurred from February 2016 to October 2017. Data were analyzed from February 2019 to July 2021.

**Interventions:**

The intervention was a home hazard removal program delivered by an occupational therapist in the home that included a comprehensive clinical assessment and a tailored hazard removal plan. Usual care control consisted of annual assessments and community referral.

**Main Outcomes and Measures:**

The primary outcome was the hazard of a fall over 12 months. Prespecified secondary outcomes included the rate of falls over 12 months, daily activity performance, falls self-efficacy, and self-reported quality of life.

**Results:**

A total of 310 participants (mean [SD] age, 75 [7.4] years; 229 [74%] women; 161 Black participants [52%]) were randomized, with 155 participants assigned to the intervention and 155 participants assigned to usual care. Retention was 127 participants (82%) in the intervention group and 126 participants (81%) in the control group. There was no difference for our primary outcome of fall hazard (hazard ratio, 0.90; 95% CI, 0.66-1.27). There was a 38% reduction in the rate of falling in the intervention group compared with the control group (relative risk, 0.62; 95% CI, 0.40-0.95; *P* = .03). At 12 months, the rate of falls per person-year was 1.5 (95% CI, 1.32-1.75) in the intervention group and 2.3 (95% CI, 2.08-2.60) in the control group. There was no difference in daily activity performance (adjusted difference, −0.20; 95% CI, −0.95 to 0.55; *P* = .60), falls self-efficacy (adjusted difference, −0.12; 95% CI, −1.25 to 1.01; *P* = .84), or quality of life (adjusted difference, 0.84; 95% CI, −0.95 to 2.64; *P* = .35).

**Conclusions and Relevance:**

This randomized clinical trial found that a brief home hazard removal program did not reduce the hazard of falls among community-dwelling older adults at high risk for falling. The intervention was effective in achieving a reduced rate of falls, a prespecified secondary outcome. This effectiveness study has the potential for delivery through the national aging services network.

**Trial Registration:**

ClinicalTrials.gov Identifier: NCT02392013

## Key Points

**Question:**

What is the effect of a home hazard removal program on hazard of a fall in community-dwelling older adults at risk for falling?

**Findings:**

This randomized clinical trial included 310 older adults. There was no difference in the primary outcome, risk of falling, between the intervention and control groups. The intervention group had a 38% reduction in falls, a secondary outcome, compared with usual care.

**Meaning:**

This randomized clinical trial found that this low-cost behavioral intervention did not result in a significant difference in risk of falling or the number of home hazards removed.

## Teaser

This randomized clinical trial examines the effectiveness of a home hazard removal program in reducing risk of falls among older adults.

## Introduction

Falls remain the leading cause of injury, long-term disability, premature institutionalization, and injury-related mortality in the older adult population.^1,2,3,4,5^ Falls are the most common cause of traumatic brain injury and fracture for older adults^6^ and can result in serious complications, such as functional dependence and fear of falling.^7,8^ Every 20 minutes, an older adult dies from the consequences of a fall.^9^ Falls are costly: $30 billion is spent treating older adults annually for the effects of falls.^10^ Most falls occur in the home,^1,11,12^ and measurable home hazards are associated with an increased risk of falling.^13^

Home hazard removal has been shown to reduce falls by 39% among people at high risk of falls in studies conducted in Europe and Australia.^14,15,16^ Interventions can include installation of grab bars, slip-resistant surfacing, and improved lighting. Home hazard removal interventions are strongly recommended by the American and British Geriatrics Societies to prevent falls^17^ but are not standard practice in the US, and the effectiveness of such programs in the US is unknown. The most recent US Preventive Services Task Force recommendations for interventions to prevent falls excluded home hazard removal, citing a lack of evidence.^18^

Therefore, the purpose of this pragmatic effectiveness and implementation study was to determine whether a home hazard removal program (HARP), adapted for delivery in the US aging services network, is effective in reducing falls. We considered this trial pragmatic because it was designed to evaluate the effectiveness of interventions in real-life routine practice conditions. We tested the hypothesis that older adults who received the program would have a reduced hazard of falling, lower rate of falls, and improved self-efficacy, daily activity performance, and quality of life compared with usual care, and we explored the costs associated with the intervention.

## Methods

### Trial Design

This hybrid randomized clinical trial^19^ adhered to the Consolidated Standards of Reporting Trials (CONSORT) reporting guideline. This study reports the effectiveness results. The trial protocol has been published previously^20^ and is available in Supplement 1. The Washington University in St Louis institutional review board approved the study, which was conducted in urban St Louis, Missouri, from January 2015 to September 2016, when the last enrolled participant completed follow-up. The design included a parallel group, prospective, randomized clinical trial with blinded assessment and superiority design. Participants were randomly assigned in a 1-to-1 ratio to receive the HARP or usual care. The HARP intervention contractor was paid with grant funding. All interventions and data collection occurred in participants’ homes.

### Participants

This randomized clinical trial was designed to measure whether HARP was effective when used in usual conditions of care. Participants for whom the intervention would be targeted in the real world were recruited. They included community-dwelling older adults at risk for falling who received services from an Area Agency on Aging (AAA).^21^ The St Louis Area Agency on Aging serves older adults and persons with disabilities (aged 18-59 years) who reside within the city of St Louis, Missouri. The agency provides a variety of programs, including nutrition programs, transportation, and information and referral, to 10 500 unduplicated individuals (or about 23% of older adults and people with disabilities in the city).

The intentionally broad inclusion criteria were aged 65 years or older, self-report of 1 or more falls in the preceding 12 months or self-report as worried about falling, and currently receiving services from an AAA. Individuals who lived in an institution or were severely cognitively impaired and unable to follow directions or report falls (as determined by a score >10 on the Short Blessed Test, a score indicative of dementia)^22^ were excluded. Potential participants were identified from the roster of more than 1300 St Louis AAA clients. Participants were selected using a random number sequence generator, contacted by phone, and invited to participate. Study flyers were distributed through a neighboring AAA.

### Randomization

Participants were allocated using a 1-to-1 ratio via randomization sequences generated a priori using a computerized formal probability model. Randomization was stratified by self-reported sex (male or female) and age. An electronic interface concealed group assignment until enrollment. Study staff not involved in data collection implemented randomization.

### Interventionists

Seven occupational therapy (OT) practitioners (including M.K. and E.S.) were trained as interventionists through 4 hours of didactic training. Interventionists were certified annually via a written and performance-based assessment.

### Intervention

The manualized intervention, adapted from Cumming and colleagues,^23^ is based on a competence/press theoretical framework.^24^ The essential component of the intervention is home hazard identification and removal. This includes comprehensive assessment of the individual, their behaviors, and the environment; a home hazard removal plan; remediation of hazards; and a booster session 6 months after the intervention. The primary mechanisms for resolving barriers in HARP are minor home repair (eg, grab bars), adaptive equipment, task modification, and education. Active components of the intervention include standardized tailoring^25^ using shared decision-making^25,26^ (so older adults control changes in their homes), self-management strategies to improve awareness of fall risks (to bring awareness of fall hazards),^27^ and motivational enhancement strategies to improve acceptance of the program.^28^ The manual includes scripted algorithms. A visit-by-visit checklist^29^ was used to record each element delivered.

During the first session (80 minutes), environmental hazards and unsafe behaviors were identified using the Westmead Home Safety Assessment.^30^ A tailored barrier removal plan was developed and implemented. In session 2 (40 minutes), the interventionist facilitated home modifications (eg, installing no-skid tape in the bathtub, engaging a building contractor to install handrails or grab bars that were provided as part of the study intervention). A third visit (30 minutes) was scheduled if needed to complete installation and training. The first 2 or 3 visits were accomplished in a mean (SD) of 3.9 (3.5) weeks. Six months after the initial visit, participants received an in-person booster session. The goals of the booster session were to identify and remediate any new home hazards and to address any issues from the initial set of strategies and modifications. No cointerventions were restricted. The intervention is described briefly in the Trial Protocol in Supplement 1 and in detail in the manual, available from the authors.

Because this trial was designed to determine whether an intervention could improve current practice, participants in the control group received usual care. Usual care in the AAA includes an annual assessment and referrals to community services (eg, medication review, fall education, minor home repairs).

### Intervention Adherence

Adherence was calculated as visits completed and the proportion of recommended modifications used of those recommended. We considered participation in 2 sessions and adherence to at least 80% of the hazard removal recommendations as necessary to achieve a treatment effect.

### Blinding

A rater (including M.K., E.S. or Y.-L.H.), blind to intervention and group allocation, conducted all baseline assessments and phone interviews. Baseline assessment raters did not conduct follow-up assessments. All raters were certified to conduct assessments after demonstrating 95% accuracy compared with a criterion-standard example.

### Outcomes

The prespecified primary outcome was the number of days to first fall in 12 months (fall hazard). Fall risk scores are a sum of dichotomized variables (1, risk; 0, no risk using evidence-based cutoff score) of known fall risk factors, including gait and balance (measured by the Performance Oriented Mobility Assessmen^31,32^), a task-oriented assessment (fall risk <25^33^), medication (prescription medications and dosages were obtained; fall risk ≥4 medications^34^), depression (assessed using the Geriatric Depression Scale Short Form,^35^ a 15-item self-report questionnaire where total scores range from 0-15 and scores of ≥5 indicate probable depression; fall risk ≥5^36^), alcohol use (assessed using the Short Michigan Alcoholism Screening Test Geriatric Version,^37^ a 10-item interview; fall risk ≥2^34,37,38^), function (assessed using Older Adult Resources Services Activity of Daily Living [OARS ADL] scale,^39^ which has respondents rate their ability to perform 14 activities on a 0-2 scale, with higher scores indicating greater independence; fall risk >4^40^), and home hazards (assessed using the Westmead Home Safety Assessment, which identifies 72 physical and environmental home hazards of older adults at risk of falling, with each item on the assessment form rated as a hazard or not a hazard, and information on all categorized hazards is identified and summarized; fall risk ≥4 hazards^41^). The trial protocol (Supplement 1) identified 4 prespecified secondary outcomes: rate of falls per person per 12 months, daily activity performance at 12 months, falls self-efficacy over 12 months, and self-reported quality of life at 12 months. Health care utilization was monitored during the study. Falls were defined as unintentional movements to the floor, ground, or an object below knee level.^42^ Fall surveillance occurred for 12 months through a daily calendar-journal tailored with family birthdays and anniversary dates for enhanced reporting accuracy.^43^ Participants reported their health care utilization. Participants returned calendars and questionnaires monthly and received a $5 gift card incentive. Participants who did not return the calendar-journal or reported a fall were contacted by phone. An interview was conducted with the participant to verify falls and determine whether the fall was injurious.

Other self-reported secondary outcomes were collected at baseline and 12 months via phone. Daily activity performance was assessed using the OARS ADL Scale.^39^ The OARS ADL Scale is a 14-item questionnaire involving 7 basic ADL items and 7 instrumental ADL items, rating participants on their ability to perform the activities independently. A person’s score can range from 0 to 28, with higher scores indicating more independence.^44^ Self-efficacy performing daily activities without falling was ascertained using the Falls Efficacy Scale-International Short Form,^45^ which measures confidence performing 10 daily activities without falling. The score is the sum of items rated 1, indicating very confident, to 10, no confidence at all. Health-related quality of life was ascertained using the 36-Item Short Form Survey.^46^

### Sample Size

We assumed that approximately 61% of control group participants would fall during 1 year of follow-up. The sample size calculation was based on the effect of a previous Australian home hazard removal study.^45^ Assuming a 20% attrition rate, a 2-sided log-rank test with an overall sample size of 300 participants (150 in each group) achieves 84% power at a .05 significance level to detect a difference of 0.19 between 0.39 and 0.58, the proportions without falls in the control and intervention groups, respectively.

### Cost Analyses

Exploratory cost analyses were conducted using self-reported health care utilization, categorized as emergency department (ED) visits, inpatient hospitalization (number of nights), inpatient therapy (occupational therapy [OT] or physical therapy [PT]), physician visits, and outpatient therapy (OT or PT), multiplied by mean costs per utilization category. The mean costs for each category were generated from the Medical Expenditure Panel Survey (MEPS), pooling data from 2012 to 2015 and inflating all costs to 2015 dollars. Mean costs per utilization category were obtained for the subset of MEPS respondents who were aged 65 years or older and reported that their health limited them performing moderate activities or that they felt they were able to accomplish less owing to physical problems. This yielded a sample of 4480 individuals who approximate the population of the current study. There are no questions specifically about fall risk or fear of falling in the survey.

Utilization data collected in this study included ED use, inpatient hospitalization, physician visits, and OT and PT visits as an inpatient and outpatient. Because it is not possible to separate therapy visits that occur as an inpatient from overall inpatient costs in MEPS, these costs were estimated manually by assuming such charges would be at least as high in an inpatient setting as in an outpatient setting. The inpatient hospitalization values were separated (into OT or PT services and all other services) and used to estimate the cost of the reported utilization by category as recorded in this study. Costs for the intervention included OT and contractor time, equipment, and materials. OT time costs were calculated using estimated reimbursement rates for evaluation and re-evaluation. A standard return-on-investment (ROI) calculation was then performed.

### Statistical Analysis

This trial used an intention-to-treat approach. For the primary outcome of time to first fall, we compared the effectiveness of the intervention to usual care using Kaplan-Meier curves with a log rank test. The Cox proportional hazard model was used to estimate the hazard ratios (HRs) associated with the intervention effect. Time 0 was the date on which the intervention was completed for the intervention group and date of baseline assessment for the control group. The ending time was time to first fall for those who experienced a fall, and 365 days after the intervention for those who did not (censored time). The secondary outcome of number of falls was examined by calculating the incidence rate ratio using a negative binomial (NB) regression model, adjusting for differences in baseline characteristics. Unlike a Poisson model, NB does not assume equality of means and variance. We evaluated the NB dispersion parameter to determine the appropriateness of the model. Model goodness-of-fit was examined by deviance and its degrees of freedom and compared with the appropriate χ^2^ distribution. Additional secondary outcomes were compared between groups by 2-sample *t* test and by linear regressions to adjust for baseline measures and other relevant variables.

Analysis was conducted using SAS statistical software version 9.4 (SAS Institute). *P* values were 2-sided, and statistical significance was set at *P* = .05. Data were analyzed from February 2019 to July 2021.

## Results

### Participant Flow

**Figure 1.**
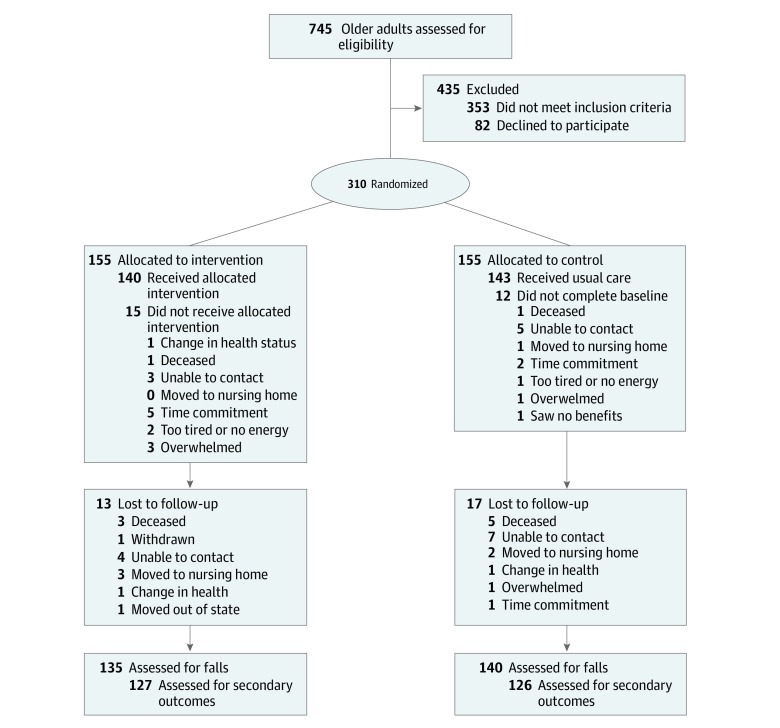
Screening, Randomization, and Follow-up of Participants

Of 745 participants assessed for eligibility, 310 were randomized, with 155 assigned to the intervention group and 155 to the control group. The 12-month retention rate was 127 participants (82%) for the intervention group and 126 participants (81%) for the control (Figure 1).

### Baseline Characteristics

At baseline, the mean (SD) age was 75 (7.4) years, 243 (78%) were women, and 170 (56%) were Black. The groups were similar in all measures (Table 1), except the intervention group was more impaired with regard to gait and balance. There were no statistically significant differences between groups for baseline fall risk scores. There was no statistically significant difference between groups for any demographic characteristics for those who did and did not complete the 12-month follow-up. The 7 interventionists had a mean (SD) of 1.3 (3.0) years of experience. All interventionists were women; 6 interventionists were White, and 1 interventionist was Black.

**Table 1.** Baseline Demographics, Covariates, and Secondary Outcomes

Variable	Mean (SD)	
	Control (n = 155)	HARP (n = 155)
Age, y	74.7 (7.4)	75.1 (7.7)
Sex, No. (%)		
Women	122 (79)	122 (78)
Men	33 (21)	34 (22)
Black race, No. (%)	83 (55)	87 (56)
Widowed, No. (%)	53 (36)	60 (44)
Education, y	13.5 (3.5)	13.6 (2.8)
Live with someone, No. (%)	115 (78)	111 (79)
Cognitive dysfunction^a^	2.63 (2.62)	3.22 (2.75)
Previous falls	1.8 (4.9)	1.6 (2.4)
Falls behavior	2.8 (.4)	2.8 (.4)
Fall risk score^b^	3.5 (1.2)	3.6 (1.2)
Gait and balance	19.8 (5.8)	17.8 (6.1)
No. of medications	7.97 (4.85)	7.2 (4.95)
Depression	2.9 (2.6)	3.5 (2.9)
Alcohol use	0.27 (.95)	0.18 (.79)
ADL performance	21.9 (4.2)	20.9 (4.5)
Home hazards	16.1 (11.7)	10 (6.7)
Falls efficacy^c^	15.8 (6.3)	16.3 (6.)
Global health^d^		
Physical functioning	39.2 (30.7)	33.9 (28.2)
Role limitation due to physical health	55.1 (30.0)	49.3 (30.5)
Bodily pain	52.5 (26.9)	50.9 (29.7)
Role limitation due to mental health	78.9 (27.6)	78.2 (29.4)
Energy and fatigue	49.8 (23.6)	43.97 (23.4)
Emotional well-being	75.1 (19.6)	72.3 (19.3)
Social functioning	67.0 (30.5)	63.2 (33.9)
General health perceptions	39.1 (24.0)	34.4 (24.4)

Abbreviation: ADL, activity of daily living.

^a^ Measured with the Short Blessed Test of memory and concentration; a score of 10 or greater indicates cognitive impairment.

^b^ Fall risk scores are a sum of dichotomized variables (1, risk; 0, no risk using evidence-based cutoff score) of known fall risk factors including gait and balance measured by the Performance Oriented Mobility Assessment,^31,32^ a task-oriented assessment (fall risk <25^33^), medication (prescription medications and dosages were obtained; fall risk ≥4 medications^34^), depression (The Geriatric Depression Scale Short Form,^35^ a 15-item self-report questionnaire where total scores range from 0-15 and scores of 5 or more indicate probable depression; fall risk ≥5^36^), alcohol use (The Short Michigan Alcoholism Screening Test Geriatric Version,^37^ a 10-item interview; fall risk ≥2^34,37,38^), function (Older Adult Resources Services ADL scale^39^ has respondents rate their ability to perform 14 activities on a 0-2 scale, with higher scores indicating greater independence; fall risk >4^40^), and home hazards (Westmead Home Safety Assessment, which identifies 72 physical and environmental home hazards of older adults at risk of falling, with each item on the assessment form rated as a hazard or not a hazard, and information on all categorized hazards identified and summarized; fall risk ≥4 hazards^41^).

^c^ Assessed with the Falls Efficacy Scale-International Short Form^45^ has respondents rate their concerns about doing the activity without falling on a scale from 1 (not at all) to 4 (very concerned). Total FES-ISF score is the sum of scores, with higher scores indicating greater fear of falling (fall risk >10).^47^

^d^ Assessed with the 36-Item Short-Form Health Survey,^18^ which assesses respondents’ quality of life.

### Adherence to Study Protocol

The mean (SD) dose of time received by intervention participants was 150.3 (29.1) minutes for the initial intervention and 108.3 (25.4) minutes for the booster session. In the intervention group, 140 participants (90%) completed the initial intervention, and of those completers, 130 participants (93%) completed the booster session. Adherence was measured by number of home modifications in use at 6 and 12 months. Self-reported adherence was 92% at 6 months and 91% at 12 months. The most frequent hazards addressed were lack of hand support, lack of adaptive equipment, tripping hazards, clutter, and lighting. The mean (SD) number of hazards for the intervention group was 10.0 (6.7) at baseline, reduced to 4.5 (5.0) at the 6-month booster session. At the 6-month booster, 54 participants (44%) in the intervention group reported implementing additional changes to their homes or routines to reduce their chance of falls. Interventionists were monitored using a visit-by-visit checklist. Fidelity to the intervention, based on a self-reported checklist completed by the interventionist, was 99%. No crossover occurred between conditions.

### Outcomes

**Figure 2.**
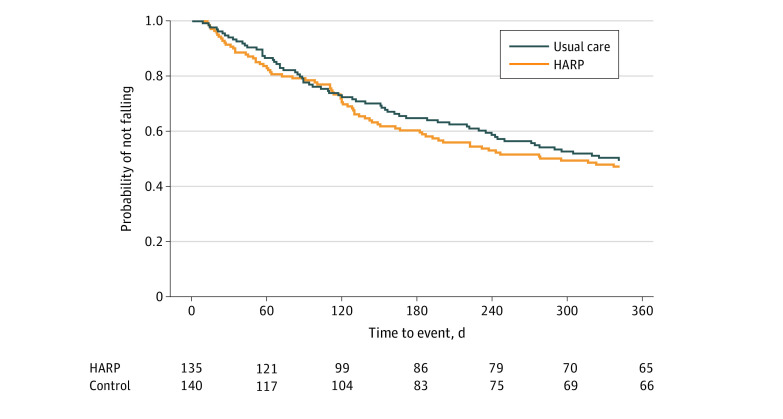
Time to First Fall by Treatment Group Participants in the intervention group had no statistically different hazard of falling compared with the control group (hazard ratio = 0.9, 95% CI, 0.66-1.27; *P* = .59). HARP indicates home hazard removal program.

At 12 months, a total of 140 participants (51%) had fallen at least once, including 67 participants (50%) in the intervention group and 74 participants (53%) in the control group. Participants in the intervention group had no statistically different hazard of falling compared with the control group (HR, 0.90; 95% CI, 0.66-1.27). Survival curves according to group assignment are presented in Figure 2.

A significant intervention effect was detected for the prespecified secondary outcome of fall rate at 12 months. The unadjusted rate of falls was 1.50 (95% CI, 1.32-1.75) falls per person per 12 months in the intervention group and 2.30 (95% CI, 2.08-2.60) falls per person per 12 months in the control group. The model (adjusted for balance and strength) showed a significant difference in rate of falls (relative risk, 0.62; 95% CI, 0.40-0.95; *P* = .03) at 12 months. The dispersion parameter was estimated at 2.45 with an SE of 0.31, Wald χ^2^_1_ of 62.5, and *P* < .001, indicating that the NB model was the preferred model to the Poisson model. The model deviance was 245.9 with 265 *df* (*P* = .80). Fall outcomes are presented in Table 2.

**Table 2.** Falls in the Control and Intervention Groups During the 12-Month Trial

Outcome	Control	HARP	RR (95% CI)
Total, No.			
Sample	140	135	NA
Falls	316	201	NA
Person-years	135.78	131.59	NA
Rate of falls, No. (95% CI)			
Per person-years	2.3 (2.1-2.6)	1.5 (1.3-1.8)	0.66 (0.55-0.78)^a^
Adjusted for baseline risk	1.01 (0.60-1.70)	0.63 (0.38-1.08)	0.62 (0.41-0.95)^b^
Persons, No. (%)			
With at least 1 fall	74 (53)	67 (50)	0.94 (0.74-1.18)^c^
With 2 or more falls	46 (33)	39 (29)	0.88 (0.62-1.25)^c^
With 3 or more falls	27 (19)	22 (16)	0.85 (0.51-1.41)^c^
With 10 or more falls	6 (4)	2 (2)	0.34 (0.07-1.68)^c^

Abbreviations: HARP, Home Hazard Removal Program; NA, not applicable; RR, relative risk.

^a^ From Poisson regression model.

^b^ From negative binomial regression model.

^c^ From 2-by-2 table Wald CIs.

For the daily activity score (OARS) total, both groups had similar baseline scores (mean [SD] score, HARP: 20.9 [4.5]; control: 21.9 [4.2]). The difference in score between the intervention group and control group was −0.91 (95% CI, −1.97 to 0.14) points in an unadjusted comparison (*P* = .09). After adjusting for baseline scores, the score difference between the intervention group and the control group was −0.16 (95% CI, −0.94, 0.61) points (*P* = .68); after full adjustment (baseline, age, sex, and race), the score difference was −0.20 points (95% CI, −0.95 to 0.55; *P* = .60). For 2 other secondary outcomes, falls self-efficacy (adjusted difference, −0.12; 95% CI, −1.25 to 1.01; *P* = .84) and quality of life (adjusted difference, 0.84; 95% CI, −0.95 to 2.64; *P* = .35), no significant difference was observed (Table 3).

**Table 3.** Secondary Outcomes at Baseline and 12 Months

Outcome	Mean (SD)				Simple comparison		Adjustment for baseline		Full adjustment^a^	
	Intervention		Control		Difference	*P* value	Difference	*P* value	Difference	*P* value
	Baseline	12 mo	Baseline	12 mo						
Daily activity performance^b^	21.00 (4.40)	21.18 (4.40)	22.02 (4.23)	22.10 (4.14)	−0.91 (−1.97 to 0.14)	.09	−0.16 (−0.94 to 0.61)	.68	−0.20 (−0.95 to 0.55)	.60
Falls self-efficacy^c^	16.29 (5.95)	14.96 (6.23)	15.80 (6.24)	14.59 (5.93)	0.37 (−1.13 to 1.88)	.62	−0.16 (−1.32 to 1.00)	.78	−0.12 (−1.25 to 1.01)	.84
Health-related quality of life^d^	41.81 (10.98)	42.26 (11.01)	44.44 (10.38)	43.74 (11.10)	−1.47 (−4.21 to 1.26)	.29	0.83 (−0.96 to 2.62)	.36	0.84 (−0.95 to 2.64)	.35

^a^ Full adjustment includes baseline characteristics, age, sex, and race.

^b^ Measured using Older Americans Resources and Services scores. Scores range from 0 to 28, with higher scores indicating more independence in daily activities.

^c^ Measured using the Falls Efficacy Scale–International Short Form,^45^ which has respondents rate their concerns about doing the activity without falling on a scale from 1 (not at all) to 4 (very concerned). Total FES-ISF score is the sum of scores, with higher scores indicating greater fear of falling (fall risk >10).^47^

^d^ Assessed with the 36-Item Short-Form Health Survey, ^18^ which assesses respondents’ quality of life.

A mean (SD) of $765.83 ($298.27) per participant was spent on delivering the intervention. The per-person declines in health care utilization were valued at $1613.63, based on MEPS data, for a 111% ROI. There were no adverse events reported during the trial.

## Discussion

This randomized clinical trial of a brief program focused on removing home hazards and teaching self-management strategies to prevent falls found that the intervention did not reduce the hazard of falling compared with the control group. However, the program did reduce the rate of falls among community-dwelling older adults by 38%. Exploratory cost analysis demonstrated that the intervention was potentially cost-effective for this high-risk population, with a 111% ROI in health care savings. The secondary outcomes of daily activity performance, falls self-efficacy, and quality of life were not significantly different.

Of 7 published randomized clinical trials focusing on home hazard removal, results have been equivocal.^48,49,50^ Although 4 studies have demonstrated a reduction in falls for community-dwelling older adults at high risk,^23,48,51,52^ 3 were unable to demonstrate a significant effect on falls.^15,53,54^ In a 2008 retrospective analysis of the characteristics of the interventions that were effective, Clemson et al^49^ identified 4 key elements from positive trials: comprehensive assessment of individual and environmental characteristics, standardized assessments, consideration of the performance of the individual within the home, and follow-up and support. The elements of positive trials were integrated into the HARP used in this study. Additional strategies (tailoring,^25,26^ self-management,^27^ and motivational enhancement^28^) were also integrated into HARP. While not definitive, this trial adds to the growing home hazard removal literature suggesting that a tailored approach using behavioral strategies and environmental supports can be effective in reducing the rate of falls and is feasible in the US.

To our knowledge, this is the first US trial of a HARP and is the first to target implementation and effectiveness. We translated the approach used by the positive Australian and European studies, where home hazard interventions are part of the health care delivery system, to the US, where they are not. Given the need to understand the effects of HARP in the US, we elected a pragmatic approach. The pragmatic elements of the study, including broad inclusion criteria, delivery in an existing aging services network, and training interventionists, did not diminish the effect of the intervention. We hypothesize that the explicit addition of the self-management component will improve the durability of the intervention, given the likelihood of functional decline expected in this population. This is supported by the report at 12 months that almost half of participants in the intervention group independently used additional fall prevention strategies. A sample representative of the AAA improves the external generalizability of the study.

Fall prevention programs, despite success in clinical trials, have not translated to a reduction in falls in the US. The most efficacious fall prevention intervention identified to date is exercise, but adherence rates are dismal in most participants.^55^ The CAPABLE trial was a home modification intervention in which an OT, a maintenance and repair professional, and a registered nurse conducted home visits to reduce disability.^56,57^ This trial measured ADLs as the primary outcome and falls self-efficacy as a secondary outcome and had positive results for both. More intervention strategies with high acceptance are needed. This trial addresses barriers to implementation in 2 ways. First, HARP demonstrated a high adherence rate for long-term effectiveness. Second, the intervention was designed for and tested in an existing national service network. Low costs and a high ROI lend support to the existing economic evaluations suggesting that home hazard removal could offer the potential to avert costs by reducing fall risks.^58^

### Limitations

This study had several limitations. Trial results were negative for the primary outcome and all secondary outcomes but one. There was no significant difference in the primary or 4 prespecified secondary outcomes. Regarding the secondary outcomes, we hypothesized that the potential mechanisms of the intervention might be related to improving daily activity performance or a reduced fear of falling; however, there were no differences in these outcomes. It is likely that the study was underpowered to detect a change, but it is also possible that these factors are not related to the types of fall risk that home hazard removal addresses. It is possible that a program focused on individual daily activity goals may be necessary to impact these secondary outcomes. There were also differences not controlled by randomization between the 2 groups with regard to previous risk of falls. The intervention group had a higher rate of previous falls at baseline than the control group. This important issue deserves further study. Second, while more than half of participants were Black, only 1 of 7 OT interventionists was Black. A better racial match to the participants would have been ideal and is a limitation. Third, participants were not blind to the outcome of the study. This single-blind study, like most fall prevention studies, is subject to this potential bias.

A possible limitation of the cost analysis and reported ROI is that the utilization data collected did not include actual costs associated with those specific events, and the utilization categories did not exactly match available cost data in the MEPS. Additionally, the cost analysis relied on self-report of health care utilization, which may not be accurately reported. The method chosen for cost analysis involved subtracting the combined costs of OT and PT visits (valued at outpatient rates) from the combined costs of inpatient hospitalizations and produced a rough estimate. However, not making this correction, ignoring the observed differences in inpatient OT and PT use, we still find an ROI of 64%. The MEPS respondents may not be identical to the study population in terms of fall risk, so the exploratory cost-effectiveness findings should be interpreted with caution.

## Conclusions

This randomized clinical trial found that a manualized HARP delivered in the community in partnership with the local AAA did not reduce the hazard of falling. However, it did reduce the rate of falls by 38%, with a potential 111% ROI. The intervention had high adherence. HARP could be an important intervention to address the growing fall rate in the US.

## Notes

Supplement 1.
Trial Protocol and Statistical Analysis Plan

Supplement 2.
Data Sharing Statement
