# Supplementary material for: Home Hazard Removal to Reduce Falls Among Community-Dwelling Older Adults: A Randomized Clinical Trial
Source: JAMA Netw Open. 2021 Aug 31;4(8):e2122044. doi: 10.1001/jamanetworkopen.2021.22044 (PMC8408671; doi:10.1001/jamanetworkopen.2021.22044)
Supplement: Supplement 1. — Trial Protocol and Statistical Analysis Plan [file jamanetwopen-e2122044-s001.pdf]

Home Hazards Removal Program (HARP)

Principal Investigator:

Susan Stark, PhD, OTR/L

Assistant Professor Occupational Therapy, Neurology and Social Work

Supported by:

Department of Housing and Urban Development

Version 1
October 1, 2014

TABLE OF CONTENTS

Page

TABLE OF CONTENTS .... **i**

1 STUDY TEAM ROSTER **5**

2 Study objectives **6**

2.1 Primary Objective 6

2.2 Secondary Objectives 6

3 BACKGROUND AND RATIONALE **6**

3.1 Background on Condition, Disease, or Other Primary Study Focus 6

3.2 Study Rationale 7

4 STUDY DESIGN **8**

4.1 Timeline and Tasks 9

5 Selection and enrollment of participants **10**

5.1 Inclusion/Exclusion Criteria 10

5.2 Study Enrollment Procedures 10

6 STUDY INTERVENTIONS **11**

6.1 Study Interventions 11

7 STUDY PROCEDURES **12**

7.1 Schedule of Evaluations 12

7.2 Overview 12

7.3 Description of Evaluations 13

7.3.1 Screening Evaluations 13

7.3.2 Enrollment, baseline, randomization 13

7.3.3 Intervention and follow up sessions 16

7.3.4 Completion/Final Evaluation 17

7.4 Process evaluation 17

7.4.1 Recruitment 17

7.4.2 Fidelity 18

7.4.3 Dose 18
7.4.4 Adherence 18
7.4.5 Safety 18
7.4.6 Cost 18
7.5 Overview of study assessments by time point. 19
8 SAFETY ASSESSMENTS 20
8.1 Safety Parameters 20
8.2 Adverse Events and Serious Adverse Events 20
8.3 Reporting Procedures 20
8.4 Follow-up for Adverse Events 20
9 INTERVENTION DISCONTINUATION 20
10 STATISTICAL CONSIDERATIONS 21
9.1 Sample Size and Randomization 21
10.3 Data Analyses 21
11 DATA COLLECTION AND QUALITY ASSURANCE 22
11.1 Data Collection Forms 22
11.2 Data Management 22
11.3 Quality Assurance 23
11.4 Quality-assurance oversight 23
11.4.1 Protocol Deviations 23
12 PARTICIPANT RIGHTS AND CONFIDENTIALITY 23
12.1 Institutional Review Board (IRB) Review 23
12.2 Study Discontinuation 24
13 PUBLICATION OF RESEARCH FINDINGS 24
14 REFERENCES 24
15 SUPPLEMENTS/APPENDICES 24

PRÉCIS

Title: Removing Home Hazards for Older Adults

Investigators: Susan Stark, PhD, OTR/L & Yan Yan, MD, PhD
Washington University School of Medicine, St. Louis, Missouri

Falls remain the leading cause of injury, long-term disability, premature institutionalization, and injury-related mortality among older adults and are projected to cost nearly $59.7 billion by 2020.[12-15, 71] Fall-related mortality increases with age.[72] Although numerous clinical interventions have demonstrated efficacy in reducing the incidence of falls,[73] translation to the community has failed; fall rates continue to escalate. Indeed, nearly one third of all adults older than 65 years fall each year. The majority of falls experienced by older adults, particularly more frail high risk older adults, occur in the home, [12, 24, 25] and measurable home hazards are associated with an increased risk of older persons falling in the United States.[74,26]

Setting. This intervention will be implemented and evaluated in the urban core of St. Louis, Missouri, an area of high need for an effective fall prevention program. In Missouri, the fall death rate for older adults is consistently higher than the national average. The rate nearly doubled between 2000 and 2009 from 38 to 72.32 per 100,000.[75] Falls are the leading cause of unintentional injury-related hospitalizations and ED visits among older Missourians; older adults account for 64% of all ED visits and hospitalizations resulting from falls, which is higher than national rates. [76, 77]

Our long-term goal is to improve older adults' ability to maintain their independence and safety in the community by translating fall prevention research into effective community programs. We plan to establish the effectiveness of a program that demonstrated a reduction in falls among community-dwelling older adults at high risk for a fall. It is a practical and scalable intervention recommended by the Centers for Disease Control and Prevention(CDC).[1] The intervention will be delivered by OTs in an Area Agency on Aging (AAA)—part of the Aging Services Network[53] in the US. We will use community-engaged research approaches[54] and a hybrid design[55] to study both the effect of the intervention in a real world setting *and* the implementation strategy. We will prepare for dissemination[56] by providing cost estimates, fidelity metrics, and intervention manuals.

Design of study:
To examine the effect of a home modification (home hazard removal) program, we will conduct a hybrid effectiveness/implementation trial of 300 older adults at risk for a fall randomized to a home hazard removal program or usual care, and follow them for 12 months. We will conduct a pragmatic randomized controlled trial/implementation study

Recruitment: We will recruit participants through the SLAAA's annual assessment of participant's health. .

Deliver intervention: Interventionists will use a manualized protocol to deliver the intervention.

Follow up: We will follow participants monthly using a highly successful calendar journal technique that has demonstrated a 99% response rate in previous studies.

Dissemination: We will disseminate the program, manuals and training programs using our advisory network.

Objectives of study:

1. *Determine the acceptability costs and feasibility of delivering the program in an Area Agency on Aging.* We will conduct a process evaluation[78] of the intervention to aid in dissemination and interpretability of the trial by evaluating acceptability, feasibility, safety, and cost. **We will test the hypothesis** that home modification interventions will have high acceptability (80% retention), high fidelity by therapists (95% of elements; 90% of dose delivered), low safety risk (no increased rate of falls compared to usual care group), and high adherence (80% of modifications in use) at 12 months.

2. Determine if the program is effective in reducing the rate and risk of falls and improving the outcome of other participant-reported outcomes (PROs) that can be effected by the intervention. **We will test the hypothesis** that older adults who receive the program will have a lower rate and risk of falls, improved self-efficacy, daily activity performance, and quality of life compared with the usual care group.

Expected outcomes: The central hypothesis of this study is that home modifications (home hazard removal) will be acceptable to older adults, practical for delivery in an existing social service network, and efficacious in reducing falls and maintaining independence for older adults. Our research team has a unique skill set to address this problem, including: a long relationship with an AAA, experience studying home modifications in the community, experience conducting pragmatic trials, and expertise in dissemination and implementation science. If successful, this study could have immediate positive impact by leading to dissemination and implementation of a program with high adherence, structured to deploy within an existing aging services network that could significantly reduce falls among community-dwelling older adults at high risk.

1 STUDY TEAM ROSTER

Researchers	Role	Phone #	Email address	alt
Susy Stark	Principal Investigator	314-932-1033	starks@wusm.wustl.edu	Fax: 314-932-1022
Emily Somerville	OT Interventionist	314-932-1027	somervillee@wusm.wustl.edu	
Marian Keglovits	OT Interventionist	314-932-1032	keglovitsm@wusm.wustl.edu	
Danielle Cobbs	OT Interventionist	314-932-1032	cobbsd@wusm.wustl.edu	
Jane Conte	Coordinator	314-932-1011	contej@wusm.wustl.edu	
Yan Yan	Statistical	314-747-1115	yany@wudoisis.wustl.edu	

Community Partners	Role	Phone#	Email address	alt
SLAAA main phone #		314-612-5918		Main fax: 314-612-5915
Dave Sykora	SLAAA Executive Director	314-657-1681	sykorad@stlouis-mo.gov	
Nora Shields-Cutler	SLAAA HealthCare Coordinator	314-657-1670	shields-cutlern@stl-mo.gov	
Anneliese Stoever	Information and Assistance Coordinator	314-657-1669	stoevera@stl-mo.gov	

2 STUDY OBJECTIVES

2.1 Primary Objective

Determine the acceptability and feasibility of delivering the program in an AAA. To examine the effect of the adapted program, we will conduct a hybrid effectiveness/implementation trial of 300 older adults at high risk for a fall who will be randomized to the adapted program or usual agency care and then followed for 12 months. We will conduct a process evaluation[78] of the intervention to aid in dissemination and interpretability of the trial by evaluating acceptability, feasibility, safety, and cost. We will test the hypothesis that home-modification interventions will have high acceptability (80% retention), high fidelity by therapists (95% of elements; 90% of dose delivered), low safety risk (no increased rate of falls compared with the usual care group), and high adherence (80% of modifications in use) at 12 months.

2.2 Secondary Objectives

Determine whether the adapted program is effective in reducing the rate and risk of falls and improving the outcome of other participant-reported outcomes that can be affected by the intervention. We will test the hypothesis that older adults who receive the program will have a lower rate and risk of falls and improved self-efficacy, daily-activity performance, and quality of life compared with the usual care group.

If successful, this study could have immediate positive impact by leading to dissemination and implementation of a program with high adherence that is structured to deploy within an existing aging services network and that could significantly reduce falls among community-dwelling older adults at high risk.

3 BACKGROUND AND RATIONALE

3.1 Background on Condition, Disease, or Other Primary Study Focus

Falls remain the leading cause of injury, long-term disability, premature institutionalization, and injury-related mortality in the older adult population.[12-15] Falls are the most common cause of traumatic brain injury and fracture for older adults,[16] and they have serious complications such as institutionalization,[17] functional dependence, and paralyzing fear of falling,[18, 19] Every 29 seconds, an older adult dies from the consequences of a fall.[20] Falls are an eminent threat to a frail, older adult's ability to maintain independence in the community. Approximately 1 in 3 community-dwelling adults aged 65 years and older fall each year,[21, 22] and those older than age 70 have an especially high fall risk.[14] Older adults who have experienced a previous fall are at a greater risk of falling again.[23] Prevalence of the home hazard condition**:** *The majority of falls experienced by older adults, particularly more frail, high-risk older adults, occur in the home*,[12, 24, 25] *and measurable home hazards are associated with an increased risk of older persons falling*

in the US.[26] Economic impact**:** Falls are costly: $30 billion a year is spent treating older adults for the effects of falls.[27] As the population ages, costs associated with falls are projected to reach $59.6 billion by 2020,[22] making fall prevention a public health priority. Gap addressed by this proposal**:** Efficacy studies conducted in Australia and Europe and results from our own efficacy trial suggest that home-modification delivered by OTs can significantly reduce falls among community-dwelling older adults at high risk.[28] Despite these findings and recommendations from the American Geriatrics Society that home modifications be routine for community-dwelling older adults at high risk for a fall,[29] home-modification implementation is not standard practice in the US. New models of care must be developed to effectively implement this strategy nationally. We plan to address this gap by implementing and testing the effectiveness of a home-hazard removal program in the US Aging Services Network. This study will be based on our recently completed home-modification trial in the US.

Home modifications to improve daily-activity performance have been studied in the US,[5, 50] and there is promising preliminary evidence of the ***efficacy*** of home-hazard removal to reduce the risk of falls (including our unpublished trial). However, no evidence shows the pragmatic ***effectiveness*** of home modifications to prevent falls in the US. Important next steps to advance the science include better understanding of the effectiveness and implementation of home-hazard removal.

3.2 Study Rationale

Fall prevention programs, despite success in clinical trials, have not translated to a reduction in falls for community-dwelling older adults in the US. The most efficacious fall-prevention intervention identified to date is **exercise**. In randomized controlled trials, multicomponent exercise groups and individually prescribed exercises have been shown to reduce the rate and risk of falls[30]. Although effective in controlled studies,[31, 32] adherence rates in community programs are low (29%–59%),[33-35] and older adults report a reluctance to initiate exercise programs to reduce their chance of falling.[36, 37] This finding is not surprising, given the low rate of participation in fitness activities among older adults in general, despite aggressive public health campaigns to increase physical activity.[38] Additional intervention strategies with high acceptance are needed to address the important public health problem of falls.

Home-modification interventions, when delivered by OTs, *also* demonstrate efficacy in reducing falls[30] among high-risk populations.[28] Home modifications can include installation of stairway railings, grab bars, slip-resistant surfacing in the bathroom, and provision of lighting. Home-hazard removal is strongly recommended by the American and British Geriatric Societies to prevent falls.[29] Indeed, home modification interventions, *when delivered by OTs,* have been shown to reduce falls by 39% among high-risk fallers[39-41] in studies conducted in Europe and Australia.

Fall-prevention interventions have not been effectively translated into prevention programs. This study will be a substantial departure from previous studies. The research proposed in this application is innovative, in our opinion, because it represents the first time an effective home-modification intervention to prevent falls will be implemented through a US social service delivery system. Successful translation into practice requires an understanding of the interaction among an intervention, the systems in which it is implemented, and the stakeholders.[42] We will use community-engaged research approaches[43-46] to determine adaptations necessary to deploy a home-modification intervention to prevent falls in the US within a reliable aging services network. We will reduce barriers to program participation by involving key stakeholders as part of the research team[47, 48] and clarifying the benefits of the research to the community.[49]

The goal of this study is to implement an evidence-based home-modification intervention for older adults that is designed to reduce the incidence of falls through a community program delivered through the aging services network with high acceptance. The program will be designed to reduce the incidence of falls among community-dwelling older adults so they may independently remain in their own homes as long as possible.

4 STUDY DESIGN

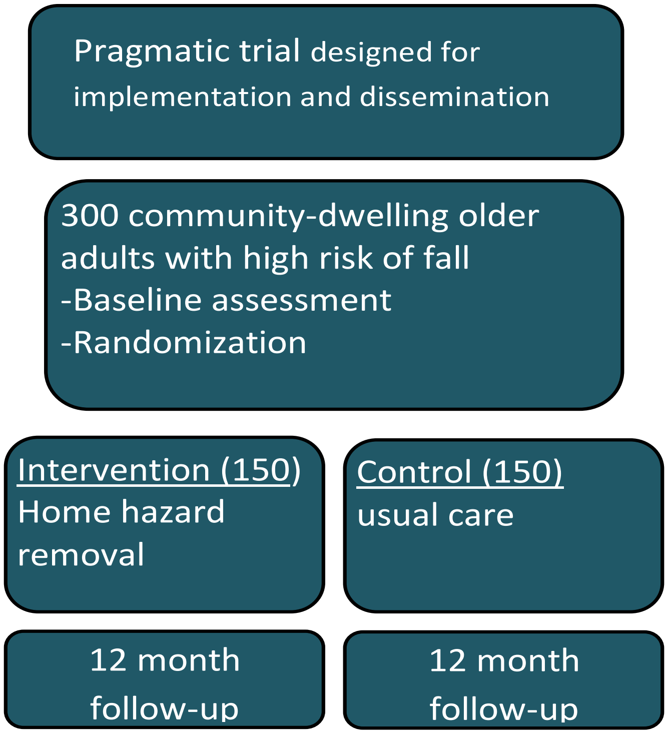

To examine the effect of a home-modification (home-hazard removal) program, we will conduct a hybrid effectiveness/implementation trial of 300 older adults at risk for a fall who will be randomized to a home-hazard removal program or usual care and then followed for 12 months. Figure 5 outlines the milestones of a pragmatic randomized controlled trial and implementation study. Study milestones are further detailed in the timeline (under Section E.1, Table 2).
Recruitment: We will recruit participants through the SLAAA's annual assessment of participants' health. We estimate more than 800 older adults will meet eligibility criteria.
Deliver intervention: Interventionists will use a manualized protocol to deliver the intervention.
Follow-up: We will follow participants monthly using a highly successful calendar journal technique that has demonstrated a 99% response rate in previous studies. Dissemination: We will disseminate the program, manuals, and training programs using our advisory network.

4.1 Timeline and Tasks

Resource	Staff	-3–0	1-6	6–12	13–18	19–24	25–30	31–36
								Month
**Personnel**								
**Weekly lab meetings**	**SS**	**xx**	**xxx**	**xxx**	**xxx**	**xxx**	**xxx**	**xxx**
**Advisory board meetings**	**SS**	**x**	▪ ▪	▪ ▪	▪ ▪	▪ ▪	▪ ▪	▪ ▪
**Staff training**	**SS**	**x**						
**Human Subjects**								
**HRPO approval/renewal**	**JC**	**x**		▪		▪		▪
**Quality Assurance Plan**								
**development**	**SS**	**x**						
**oversight**	**WU**		▪	▪	▪	▪	▪	▪
**Report to advisory committee**	**SS**		▪	▪	▪	▪	▪	▪
**Study Tasks**								
**Manuals & training materials**	**SS**		**xxx**					
**Recruit , enroll participants**	**JC**		**xxx**	**xxx**	**xxx**			
**Baseline assessment**	**ES**		**xxx**	**xxx**	**xxx**			
**Deliver intervention**	**ES**			**xxx**	**xxx**	**xxx**	**xxx**	
**Follow-up**	**JC**			**xxx**	**xxx**	**xxx**	**xxx**	**xx**
**Data management and analysis**								
**Database**	**LH**	**xxx**	**xxx**	**xxx**	**xxx**	**xxx**	**xxx**	**xxx**
**Conduct interim analyses**	**YY**			**x**	**x**	**x**	**x**	**x**
**Conduct final analyses**	**YY**							**xxx**
**Dissemination and Implementation**								
**Meeting with participants and community**	**SS**							▪
**Dissemination planning meeting**	**SS**	▪		▪		▪		▪
**Peer-reviewed publications**								
**Protocol**	**SS**		▪					
**Implementation outcomes**	**SS**							▪
**Intervention outcomes**	**SS**							▪

Note: x = 2 months; **▪ = single event;** SS=S. Stark; JC = J. Conte; WU = oversight; ES = E. Somerville; LH = L. Hu; YY = Y. Yan

5 SELECTION AND ENROLLMENT OF PARTICIPANTS

5.1 Inclusion/Exclusion Criteria

Inclusion criteria: 1) aged 65 years or older; 2) self-report of one previous fall or more in the preceding 12 months or self-report as "worried about falling;" and currently receiving services from SLAAA.
Exclusion criteria: 1) residents of nursing homes; 2) individuals with severe cognitive impairment who are unable to give consent to participate (as determined by a score of greater than 10 on the SBT for memory[10])

5.2 Study Enrollment Procedures

Study population, eligibility, and screening. The National Aging Program Information System (NAPIS) is a database of information collected annually by the AoA from state units on aging describing the general health, nutritional, financial, functional, and environmental status of an older adult. Missouri's assessment (MA4) currently ***includes an evidence-based fall-risk screening*** as part of an extensive battery of standardized assessments. In the current cohort of 1,331 older adults screened in CY 2013 by SLAAA, the average age of the cohort is 77 years, 67 percent are female and 74% are African American. More than 25% reported a fall in the past 12 months, and 40% are "worried about falling." The average score on ADL performance was 4 (range, 1–7); indicating moderate ADL impairment). We will recruit from this cohort of older adults who are "at high risk for a fall."[79] We estimate more than 800 older adults will meet eligibility criteria in the cohort. [2, 3] The SLAAA staff will identify potential participants and assist in recruiting participants at no cost

We will recruit 300 older adults, conduct baseline assessments, and randomize participants to receive the adapted program or usual care. Outcomes (falls, fall self-efficacy) will be assessed by a blinded evaluator at baseline and 12 months after intervention. Flow through the study is illustrated in Figure 5. All assessments and study visits will occur in participants' homes.

Recruitment. We will randomly select a sample of older adults from the large observational cohort of older adults assessed annually by SLAAA via NAPIS as part of service delivery[80] and invite them to participate by telephone. Refusals will be tracked for attribution of cause.

Consent and enrollment. Consent will be obtained at the baseline home assessment visit. Potential participants will be provided with a study brochure and a copy of the large-print consent form. Scripted details of the project including the randomization procedure, study interventions, and follow-up procedures will be discussed (under Section C.2.c).

Randomization sequence generation, allocation concealment, and implementation. Participants will be allocated using a 1:1 ratio via randomization sequences generated a priori using a computerized formal probability model. The allocation ratio will be maintained at periodic intervals. Groups will be balanced with regard to race (self-reported African American

or “other”) and sex. Randomization sequence concealment will be achieved by the Research Electronic Data Capture (REDCap) system.[81] After baseline assessment, the study coordinator will verify that all eligibility requirements are satisfied and then elicit treatment assignment.

6 STUDY INTERVENTIONS

6.1 Study Interventions

Control group (usual care). During the treatment phase of the study, each participant randomized to control will receive usual care from SLAAA, which includes annual NAPIS evaluation (in home) and individualized referrals to services based on identified need. Referrals are generated by case managers or SLAAA staff based on the annual assessment or if older adults (or their family members) call and request a referral for services.

Procedures for intervention (treatment). Participants who are randomized to treatment will receive usual care plus the home-modification (home-hazard removal) intervention. The intervention, based on a competence/press theoretical framework,[96] will be provided by registered and licensed OT. The intervention will be delivered via a manualized intervention using a visit by visit checklist. Home modifications[79] include a 2-hour home assessment conducted by an OT in the participant’s home using the WeHSA. A comprehensive clinical assessment will be performed on each participant to determine functional limitations that may interact with hazards in the home and result in a fall. Environmental hazards and unsafe behaviors will be identified. A barrier-removal plan will be provided to the older adult. The OT will facilitate home modifications through housing services programs and provided follow-up 2 weeks after the initial home visit to ensure that the participant adopted the recommended modifications. It is anticipated that 2-3 visits of 90 minutes each will be provided. At about 6 months participants will receive a booster session (in home visit by an OT) to reevaluate performance in the home and modify the intervention plan as needed.

Interventionist Training

Each interventionist will receive a 3 hour didactic training and additional 2 hours of guided self-directed learning.

5.2 Process Evaluation

We will evaluate a set of implementation outcomes distinct from clinical treatment outcomes[99] that are relevant for decision makers and important for implementation and dissemination of the intervention. The components and data sources of the process evaluation are presented in Table 1. We will conduct a process evaluation to understand the fidelity, safety, cost, and quality of the intervention.[100] We will monitor recruitment metrics for all eligible participants, including demographic characteristics, enrollment status, and reason for decline. We will determine the fidelity of the intervention by determining whether the intervention was delivered as planned and

calculating the dose of the intervention received by both groups (number of minutes and number of sessions delivered). OTs will rate adherence at the final visit as the number of recommendations implemented per recommendations suggested.[6] Long-term adherence will be calculated as the number of recommendations used at 12 months per recommendations suggested. We will examine reasons for abandonment of strategies to inform the final program. We will explore the safety of the intervention by determining the rate and severity of falls (calculated with an algorithm).[101] Environmental modifications are provided through a patchwork of services in the US; therefore, reliable cost data are not available. We will track the cost of service provision (in the treatment group). We will record OT time to provide services (direct and indirect)[102] and costs of equipment issued to participants. Costs of contracting services will include installation (labor and materials) and maintenance or repair during follow-up. All OT time will be recorded using current procedural terminology codes. The hourly wage of the contractor will be estimated using wage rates for the St. Louis area as reported by the U.S. Bureau of Labor Statistics.[103] The cost of all materials will be captured from invoices. Costs will be estimated from the payor perspective (e.g., Medicare allowable costs will be used to estimate covered clinical encounters).[104]

7 STUDY PROCEDURES

7.1 Schedule of Evaluations

7.2 Overview

Assessments will be conducted at baseline and 6 months after intervention for participants in the treatment group and will occur in participants' homes. For those in the control group, no 6 month assessment will occur. A 12 month phone follow up will occur for all participants.

Treatment

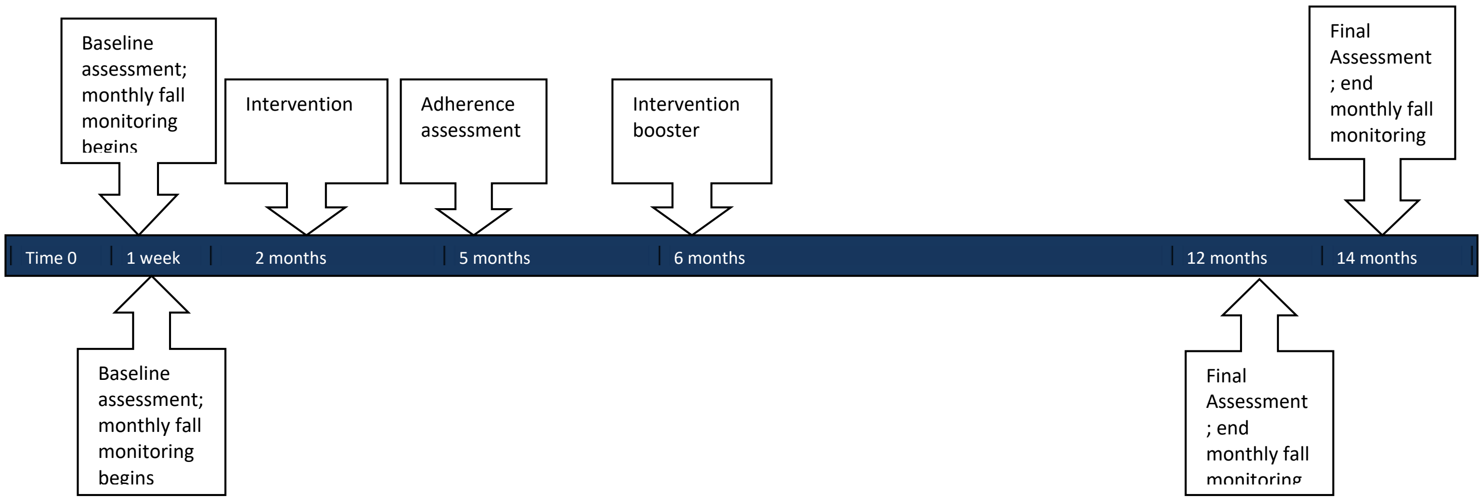

Control

7.3 Description of Evaluations

7.3.1 Screening Evaluations

7.3.1.1 Short Blessed Test (SBT). The SBT[61] will be used to screen for severe cognitive impairment. The SBT has good validity for older adults[61] and correlates well with the Mini-Mental State Exam[62]. This brief, six-item questionnaire assesses cognition, memory, and orientation. Incorrect items are scored as the product of the number of errors made and a weighting factor; the sum of incorrect item scores comprises the total score. Total scores range from 0-28; scores of 10 or more are consistent with dementia and other cognitive difficulties. Individuals who receive a SBT score 10 or more will not be allowed to participate in the proposed study.

7.3.1.2 Any self-report of fear of falling in past year.

7.3.1.3 Falls in past year. Any report of falls in the previous year will be an automatic inclusion.

7.3.2 Enrollment, baseline, randomization

7.3.2.1 Enrollment

Informed Consent Process**.** All elements of consent will be reviewed with older adults prior to enrolling in the study, including the purpose of the study, risks, benefits, alternatives to the study, how confidentiality will be maintained, the PI's contact information, no consequences to withdrawal, and how study results will be shared. All study staff have undergone IRB training. In addition, all staff members have participated in cultural competence training and are trained to interview older adults. All staff members are trained to obtain assent, which is particularly important for older adults with low vision, low literacy levels, or limited English proficiency. All study documents are available in multiple formats, are developed in 16-point Arial font, and are written in plain language. All informed consent documents are reviewed by the laboratory advisory board of older adults (involved in the design of the study) to ensure clarity for older adults.

Steps to understand the elements of informed consent. Written informed consent to participate in the study will be obtained before any test or measurement is performed. Eligibility will be confirmed prior to enrollment. All potential participants will be screened using a script by the coordinator. Screening will take place in person or over the phone. Potential participants will be given a copy of the large-print informed consent document and a study review sheet written at the appropriate reading level to review. During screening, all participants (1) will have a detailed, plain-language explanation of the study and what is expected of them; (2) will discuss potential problems that could interfere with participation; (3) will have their questions answered; and (4) will receive a plain-language summary of the study and contact information for the PI and study coordinator. Consent will occur during the first home assessment visit. The consent form will be signed by a witness and stored in the office of the PI under double locks.
Participants will be advised in the consent form that there is a possibility that their medical research record, including identifying information, may be inspected and photocopied by

officials of Federal or state government agencies and the Washington University Human Research Protection Office.

Enrollment. Enrollment is defined as the date all of the screening criteria are met and the individual agrees to participate. The enrollment date will be recorded on a case report form.

7.3.2.2 Randomization.

Participants will be allocated using a 1:1 ratio via randomization sequences generated a priori using a computerized formal probability model. The allocation ratio will be maintained at periodic intervals. Groups will be balanced with regard to race (self-reported African American or “other”) and sex. Randomization sequence concealment will be achieved by the Research Electronic Data Capture (REDCap) system.[81] The study coordinator will verify that all eligibility requirements are satisfied and then elicit treatment assignment prior to baseline assessment.

7.3.2.3 Baseline Assessment

Demographics. Demographic and other pertinent information, including assistance received, adaptive equipment usage, and falls, will be collected.

OARS ADL scale. The OARS ADL scale[51] will be used to screen for functional performance. The OARS ADL has been validated for the older adult ED population[52], is brief and easy to administer. Potential participants are asked about their ability to perform each of 14 activities. Responses are scored on a 0-2 scale, with higher scores indicating greater independence. Individuals who receive a score of 0 on one or more activities or a score of 1 on two or more activities will qualify for the study.

Falls Efficacy Scale-International (FES-I). The Falls Efficacy Scale-International (FES-I)[53] will be used to assess participants’ self-efficacy in performing daily activities without falling. The FES-I has good test-retest reliability and validity and consists of a list of 16 daily activities (e.g., getting in and out of bed) on which respondents rate their confidence doing each activity without falling on a scale from 1 (very confident) to 10 (no confidence at all). Total FES-I score is the sum of each activity score, with higher scores indicating greater fear of falling.

Patient Reported Outcomes Measurement Information System (PROMIS) Global Scale. Self-rated health will be measured using the Patient Reported Outcomes Measurement Information System (PROMIS) Global Scale[54] which measures general perceptions of health. We will use two scales: global physical health (overall physical health, physical function, pain, and fatigue) and global mental health (quality of life, mental health, satisfaction with social activities, and emotional problems). Reliability and validity of the scales have been established[54, 55].

Westmead Home Safety Assessment (WeHSA). The Westmead Home Safety Assessment (WeHSA)[57] will be used to identify the number of environmental hazards in all areas of the home (e.g., seating, bedroom, medication management) via 72 categories. Each category is specified with explicit descriptors to qualify a given hazard with a score of 0 for absent and 1 for present. Total hazards are summed.

Short Michigan Alcoholism Screening Test – Geriatric Version (S-MAST-G). The S-MAST-G[58] will be used to screen for the presence of an alcohol problem in potential participants. The S-MAST-G has been validated for the older adult population[59]. Potential participants will answer 10 yes/no questions about their alcohol consumption in the past year; two or more "yes" responses indicate a likely alcohol problem..

Geriatric Depression Scale Short Form (GDS-SF). The GDS-SF[60] will be used to assess depression levels in participants. The GDS-SF is a 15-item self-report questionnaire specifically designed and validated with the older population, and correlates highly with the original 30-item GDS (r=.84)[60]. Participants answer yes-or-no questions about their feelings in the past week. Total scores range from 0 to 15, and scores of 5 or more indicate probable depression.

Short Blessed Test (SBT). The SBT[61] will be used to screen for severe cognitive impairment. The SBT has good validity for older adults[61] and correlates well with the Mini-Mental State Exam[62]. This brief, six-item questionnaire assesses cognition, memory, and orientation. Incorrect items are scored as the product of the number of errors made and a weighting factor; the sum of incorrect item scores comprises the total score. Total scores range from 0-28; scores of 10 or more are consistent with dementia and other cognitive difficulties. Individuals who receive a SBT score 10 or more will not be allowed to participate in the proposed study.

Prescription medications. A medication inventory will be collected.

Performance Oriented Mobility Assessment (POMA). Mobility and balance will be assessed using the Performance Oriented Mobility Assessment (POMA)[63], a task-oriented gait and balance assessment that has been validated for the older adult population.

Strength, and range of motion of the upper extremity. Strength and range of motion of the upper extremity will be assessed using group muscle tests and goniometry and scored as within normal limits, within functional limits, or impaired[64]. Strength of the dominant hand will be assessed using a dynamometer.

Pelli-Robson. We will measure visual contrast sensitivity using the Pelli-Robson test[65]. We will score participants' binocular contrast sensitivity letter by letter.

Falls behavioral Scale for Older People (FaB). Protective behavioral factors to prevent falls will be assessed using the FaB. This instrument includes ten behavioral dimensions: cognitive adaptations, protective mobility, avoidance, awareness, pace, practical strategies, displacing activities, being observant, changes in level, and getting to the phone[66].

7.3.3 Intervention and follow up sessions

7.3.3.1 Intervention

Participants randomized to the intervention group will receive the intervention over 2 visits and one booster session at 6 months according to a manualized intervention. Briefly, for the intervention group, the occupational therapist will begin the intervention during the WeHSA. During the WeHSA the occupational therapist will assist the older adult to identify home hazards, work through potential solutions to reduce or remove the hazard, if possible, implement solutions (such as removing a throw rug), create plan for additional hazard removal, begin self-management training for behavioral and environmental fall risks, and provide training to complete the calendar journal. Between visits, the occupational therapist will assist the older adult to obtain equipment or home modifications. At the second visit, the occupational therapist will provide any necessary installation of equipment (such as a tub transfer bench), explain the purpose of each new equipment, teach techniques to safely use the equipment, and observe the older adult safely using the equipment. The occupational therapist will also continue the self-management training to improve daily activity performance and safety.

7.3.3.2 5 month adherence

A phone call to evaluate adherence will be made at approximately five months (in anticipation of the 6 month booster session).

7.3.3.3 Six month Booster

A 6-month booster session will also be provided to assist the older adults with any new fall hazards and to reinforce self-management/mindfulness training techniques. To identify additional fall hazards, the occupational therapist will complete the WeHSA with the older adult and make recommendations for solutions or provide additional training.

7.3.3.4 Monthly monitoring falls

To record falls, an individually customized calendar journal will be designed for each participant for daily recording of falls[48]. Our method of tailoring the calendar journal includes life event date anchors, incentives and personalization[49], we will construct the participant-tailored calendar journals[50]. There will be space to record the primary outcome of falls (fall or no fall) each day. If a participant falls, they will be instructed to complete a fall form. Calendar journal pages and fall forms will be collected monthly via U.S. mail. Falls will be verified with a follow-up telephone interview by a community coordinator.

7.3.3.5 Raters

Follow up assessments will conducted by trained raters blind to group allocation. The follow-up sessions will repeat assessments conducted at the first study home visit and first phone call.

7.3.4 Completion/Final Evaluation

List each assessment to be performed at the participant's final visit.
Falls behavioral Scale for Older People (FaB). Protective behavioral factors to prevent falls will be assessed using the FaB. This instrument includes ten behavioral dimensions: cognitive adaptations, protective mobility, avoidance, awareness, pace, practical strategies, displacing activities, being observant, changes in level, and getting to the phone[66].

OARS ADL scale. The OARS ADL scale[51] will be used to screen for functional performance. The OARS ADL has been validated for the older adult ED population[52], is brief and easy to administer. Potential participants are asked about their ability to perform each of 14 activities. Responses are scored on a 0-2 scale, with higher scores indicating greater independence. Individuals who receive a score of 0 on one or more activities or a score of 1 on two or more activities will qualify for the study.

Falls Efficacy Scale-International (FES). The Falls Efficacy Scale-International (FES-I)[53] will be used to assess participants' self-efficacy in performing daily activities without falling. The FES-I has good test-retest reliability and validity and consists of a list of 16 daily activities (e.g., getting in and out of bed) on which respondents rate their confidence doing each activity without falling on a scale from 1 (very confident) to 10 (no confidence at all). Total FES-I score is the sum of each activity score, with higher scores indicating greater fear of falling.

Patient Reported Outcomes Measurement Information System (PROMIS) Global Scale. Self-rated health will be measured using the Patient Reported Outcomes Measurement Information System (PROMIS) Global Scale[54] which measures general perceptions of health. We will use two scales: global physical health (overall physical health, physical function, pain, and fatigue) and global mental health (quality of life, mental health, satisfaction with social activities, and emotional problems). Reliability and validity of the scales have been established[54, 55].

7.4 Process evaluation

7.4.1 Recruitment

Enrollment and retention will be tracked by the research coordinator using REDCap, an online data entry program. Recruitment information for each participant will include the potential participants' initials, gender, birthdate, race, zip code, stroke date, admission date, and discharge date. Reasons for denying participation in the study as well as study attrition will also be tracked via REDCap.

7.4.2 Fidelity

In order to guarantee treatment fidelity, or our ability to provide the same treatment as planned to each participant, we follow similar methods of Weersing, et al[[36]] and use a Visit by Visit Treatment Grid. This grid specifically outlines the pre, during, and post treatment visit requirements for each treatment session. It is designed to be a checklist in which therapists check off the action once it is completed. During weekly interventionist meetings, the lead therapist will review the treatment grid for each participant to guarantee the necessary components of the intervention are being delivered.

7.4.3 Dose

In order to effectively measure the dose of treatment provided for each participant, we will measure both the dose that was delivered to each person (minutes of each treatment session and number of session) as well as the dose received (recommendations implemented/total recommendations). We will use a spreadsheet or Time Log to track minutes spent in each treatment session and another spreadsheet or Prescription Log to track the recommendations made and implemented for each participant.

7.4.4 Adherence

Adherence measures the participant's continued use of the implemented modifications. We will calculate adherence using the standardized approach that Cumming, et al[[37]] used: adherence = recommendations used/total recommendations. Interventionists will rate adherence with intervention at the final session by using the Prescription Log to track recommendations made, implemented and reasons that any recommendations were not implemented. Initial adherence will be a proportion of the number of recommendations implemented /recommendations suggested. Finally, we will determine the number of recommendations used/recommendations suggested at 6 months. Reasons for abandoning strategies will be examined using the Adherence Log; in which the participant will report on current level of use for each modification: very useful, somewhat useful, not at all useful, no longer use equipment. Any independence that was regained by improved sensorimotor performance will not count against the adherence rating.

7.4.5 Safety

To explore the safety of our study, we will measure the number of falls and the circumstances surrounding the falls with a self-report Fall Form used at the 6 month follow up visit. The rate and severity of the falls will be calculated using a standardized algorithm established by Tinetti, et al, 1988[[38]]. We will also track both health care utilization for participants in both groups (# of emergency room and out patient visits, # of hospitalizations, and number of days in therapy) using the Health Care Utilization Form.

7.4.6 Cost

The cost of the treatment will be measured by tracking cost of modifications and adaptive equipment for each participant. This will be tracked using the Invoice Form which includes both

costs from the contractor as well as costs of any equipment ordered from a medical supply company or obtained from a community resource (medical equipment lending program). The total amount of money spent on each person will be tracked on this form.

7.5 Overview of study assessments by time point.

	Phone Screening	Consent visit	Baseline assessment	Treatment session (tx group only)	Follow-up months 1-12	5 month phone call (tx group only)	6 month Booster in home (tx group only)	12 month phone call
Consent		x						
Calendar Questionnaire		x						
Demographics		x						
WeHSA			x	x			x	
SMAST-G			x					
GDS		x						
SBT	x							
Medication		x						
ROM			x					
Strength			x					
Grip Strength			x					
Near Visual Acuity			x					
Contrast Sensitivity			x					
POMA			x					
Falls			x	x	x			x
OARS			x					x
FES		x						x
PROMIS		x						x
Falls behavioral Scale		x						x
Fidelity to treatment (visit by visit grid)			x	x			x	
Time (Dose), Completion of Session, Cost				x			x	
Adherence						x	x	x

*randomization before T1

*both groups will send in monthly fall journal information

8 SAFETY ASSESSMENTS

8.1 Safety Parameters

To explore the safety of our study, we will measure the number of falls and the circumstances surrounding the falls and health care utilization for participants in both groups (# of emergency room and outpatient visits, # of hospitalizations, and number of days in therapy) using the Health Care Utilization Form. The rate and severity of the falls will be calculated using a standardized algorithm established by Tinetti, et al, 1988[[38]]. We will also track both health care utilization for participants in both groups (# of emergency room and outpatient visits, # of hospitalizations, and number of days in therapy) using the Health Care Utilization Form.

8.2 Adverse Events and Serious Adverse Events

An **adverse event (AE)** are defined as any unfavorable and unintended diagnosis, symptom, sign (including an abnormal laboratory finding), syndrome or disease which either occurs during the study, having been absent at baseline, or if present at baseline, appears to worsen. Adverse events are to be recording regardless of their relationship to the study intervention.

A **serious adverse event (SAE)** is generally defined as any untoward medical occurrence that results in death, is life threatening, requires inpatient hospitalization or prolongation of existing hospitalization, results in persistent or significant disability/incapacity, or is a congenital anomaly.

8.3 Reporting Procedures

All serious adverse events will be reported to the HRPO in the falling time frames: a) death – immediately; b) life-threatening within 7 calendar days; c) all other SAEs within 15 calendar days using the Electronic Serious Adverse Event Reporting System. Should there be a serious adverse event that occurs that increases the risks to the participants, the study will be stopped, an investigation will be conducted, and a findings report will be generated before the study is resumed.

8.4 Follow-up for Adverse Events

AEs will be followed until resolved or considered stable for the study period.

9 INTERVENTION DISCONTINUATION

Safety data will be collected on any participant discontinued due to an AE or SAE. In any case, every effort must be made to undertake protocol-specified safety follow-up procedures. If voluntary withdrawal occurs, the participant will be asked to continue scheduled evaluations,

complete an end-of-study evaluation, and be given appropriate therapy until the symptoms of any AE resolve or the participant's condition becomes stable.

10 STATISTICAL CONSIDERATIONS

9.1 Sample Size and Randomization

Power computations reflect two general principles. The first is that we will be conservative by underestimating and overstating power. Second, estimates of statistical power will be consistent with the method of analyses. Our primary outcome is time to event; therefore, we will determine the sample size and power within the survival data analyses framework. The Cumming et al.[79] sample is most similar to our target sample and similar regarding the method of fall ascertainment using daily calendars. On this basis, we assume that approximately 61% of control group participants will fall during 1 year of follow-up. For a meaningful reduction in fall risk, an intervention needs to demonstrate at least 30% reduction in falls.[105, 106] The Cochrane Review of interventions for preventing falls in older, community-dwelling adults reported that randomized trials of home-safety interventions and personal mobility aids reduced the rate of falls (RaR, 0.59; 95% CI, 0.42–0.82) among high-risk participants defined as having a history of falling or other risk factor for falls.[30] Another meta-analysis[28] of four randomized trials of environmental interventions, which included some of the same studies as the Cochrane Review, reported a 39% reduction in the risk of falls in high-risk populations, defined as a history of falling in the past year or presence of other risk factors for falling. Because our target sample can be defined as "high risk," and because our intervention can be defined as a high-intensity home modification, we project a 30% or greater reduction in fall risk at 12 months after treatment. Figure 6 displays the power as a function of total sample size given a fall rate of 42% (event-free rate of 0.58) in the intervention group and a fall rate of 0.61 (event-free rate of 0.39) in the control group, with a 20% attrition rate. A two-sided log-rank test with an overall sample size of 300 participants (150 in each group) achieves 84% power at a 0.05 significance level to detect a difference of 0.19 between 0.39 and 0.58—the proportions without falls in the control and intervention groups, respectively. This corresponds to a hazard ratio of 0.5785 of fall in intervention versus control groups. These results are based on the assumption that the hazard rates are proportional.

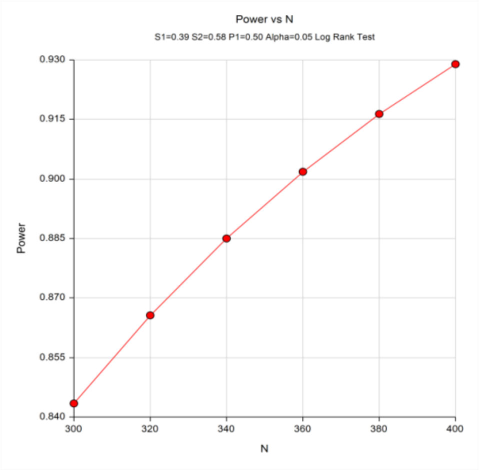

Figure. 6. Power calculation.

10.3 Data Analyses

We will examine the process data before the efficacy analyses.[107] We will use descriptive statistics to describe recruitment rates and reasons for not enrolling. <u>To explore the extent and</u>

quality of the implementation of the intervention, we will compare the characteristics of patients who complete the assigned intervention with those who do not for differences in fall risks. Adherence and cost estimates will be conducted for participants in the intervention group. Descriptive statistics will be used for adherence. We will test the hypothesis that the intervention will have high acceptability (at least 80% retention), high fidelity by therapists (at least 95% of elements and at least 90% of dose delivered), and high adherence to the program (at least 80% of modifications in use) at 12 months. These hypotheses will be tested using a 1-sample 1-sided proportion test. For costs, initial analysis will be conducted to determine what type of approach will be necessary to make unbiased and relevant estimates of the cost of the intervention. We will compare the effectiveness of the program and usual care with the time to the first fall over 12 months as the primary outcome of interest. Survival analysis techniques will be used where time zero is the date on which the intervention is completed, and the ending time is 12 months after the intervention. Participants who do not have a fall will be censored at the end of the study. Participants who move to a nursing home will be censored on the date of moving. For those who fall during follow-up, the time to the first fall will be the event time. Kaplan-Meier product limit estimates will be used to describe the fall experiences for the intervention and control groups, with the log-rank statistic testing for significant difference. Prespecified covariates (e.g., age, race, sex, number of reported medications, mobility, ADL scores, and visual acuity) will be adjusted using the Cox proportional hazards model. We will test the interventions effect using the Cox proportional hazards model and adjusting for covariates. The regression coefficient of the indicator variable quantifies the difference in the log hazard of time to first fall between intervention and control groups. We will determine whether fall-prevention treatment is superior to usual care on secondary outcomes (total number of falls, number of injurious falls) and participant-reported outcomes (daily-activity performance, fall self-efficacy, and global health). We expect that the intervention group will experience fewer total falls, fewer injurious falls, greater daily-activity performance, greater self-efficacy, and greater quality of life compared with the usual care group.

11 DATA COLLECTION AND QUALITY ASSURANCE

11.1 Data Collection Forms

Data will be collected at each follow up point by a blinded rater over the phone. Data will be directly entered into an online data management software (see 11.2) Back up paper forms will be available (See MOP).

11.2 Data Management

Data-entry and data-management systems have been built to ensure accurate and complete data sets. Data will be entered via Web-based screens uploaded to a secure server. REDCap provides secure, Web-based applications, including real-time validation rules with automated data type and range checks at the time of data entry[81]. Data will be automatically backed up

daily, and copies will be stored offsite. All paper data forms will be reviewed weekly by the laboratory manager to ensure complete and accurate entry. Incomplete data will be returned to staff to rectify the situation by data edits, chart reviews, participant contact, or search of the National Death Index to ascertain status.

11.3 Quality Assurance

Training. Examples of quality assurance mechanisms include trained and certified field personnel,[108]; comprehensive documentation of operations and procedures,[109] valid and accurate instrumentation (see "Outcomes and assessments"), field-tested data collection forms from our current HUD-funded study, a data-entry system with validation and checks[81] with automated systems for scheduling and reminders, and quarterly audits of the database.

11.4 Quality-assurance oversight

QA oversight will be provided by the Washington University Human Research Quality Assurance (HRQA) program. The program will monitor this study to ensure the protection of human subjects and confirm that research is conducted in compliance with Federal regulations and university policies. In addition, study staff members (Conte and Somerville) have undergone intensive training in quality assurance, quality control, ethics, and operations through the HRQA program. The PI and the study team will have access to continuing education, consultation, and quality-assurance procedures and forms. Quality assurance will ensure the successful activation of the research; the efficient and accurate collection of data to meet protocol objectives; and monitoring and auditing trial progress to ensure compliance with regulations, standards of operation, and subject safety.

11.4.1 Protocol Deviations

Protocol deviations will be captured on a protocol deviation forma and reviewed by the study team and advisory board quarterly.

12 PARTICIPANT RIGHTS AND CONFIDENTIALITY

12.1 Institutional Review Board (IRB) Review

This protocol and the informed consent document (Appendix) and any subsequent modifications will be reviewed and approved by the HRPO committee responsible for oversight of the study.
Informed Consent Forms

A signed consent form will be obtained from each participant. For participants who cannot consent for themselves, such as those with a legal guardian (e.g. person with power of attorney), this individual must sign the consent form. The consent form will describe the purpose of the study, the procedures to be followed, and the risks and benefits of participation. A copy will be given to each participant or legal guardian and this fact will be documented in the participant's

record. we answered ‘no’ in myIRB questions on individuals lacking compacity to consent Is this regarding folks who can’t physically sign but understand?

12.2 Participant Confidentiality

Any data, forms, reports, photographs, and other records that leave the site will be identified only by a participant identification number (Participant ID, PID) to maintain confidentiality. All records will be kept in a locked file cabinet. All computer entry and networking programs will be done using PIDs only. Information will not be released without written permission of the participant, except as necessary for monitoring by IRB or HUD.

12.3 Study Discontinuation

The study may be discontinued at any time by the IRB or government agencies as part of their duties to ensure that research participants are protected.

13 PUBLICATION OF RESEARCH FINDINGS

Publication of the results of this trial will be governed by the policies and procedures developed by the Steering Committee. Any presentation, abstract, or manuscript will be made available for review by the sponsor.

14 REFERENCES

Provide the citations for all publications and presentations referenced in the text of the protocol.

15 SUPPLEMENTS/APPENDICES

15.1 Redcap data entry screen forms

15.2 IRB approval
