# Supplementary material for: Home Hazard Removal to Reduce Falls Among Community-Dwelling Older Adults: A Randomized Clinical Trial
Source: JAMA Netw Open. 2021 Aug 31;4(8):e2122044. doi: 10.1001/jamanetworkopen.2021.22044 (PMC8408671; doi:10.1001/jamanetworkopen.2021.22044)
Supplement: Supplement 2. — Data Sharing Statement [file jamanetwopen-e2122044-s002.pdf]

Data Sharing Statement

Stark. Home Hazard Removal to Reduce Falls Among Community-Dwelling Older Adults. *JAMA Netw Open*. Published August 31, 2021.
doi:10.1001/jamanetworkopen.2021.22044

Data
Data available: No
